# Evaluation of Testicular Volume and Correlation with Sperm Production in Martina Franca Donkeys: A Parameter to Consider When Approving Breeding Jacks

**DOI:** 10.3390/ani13233619

**Published:** 2023-11-23

**Authors:** Roberta Bucci, Ippolito De Amicis, Salvatore Parrillo, Domenico Robbe, Augusto Carluccio

**Affiliations:** Department of Veterinary Medicine, University of Teramo, Piano d’Accio, 64100 Teramo, Italy; ideamicis@unite.it (I.D.A.); drobbe@unite.it (D.R.); acarluccio@unite.it (A.C.)

**Keywords:** biodiversity, endangered species, testicular volume, sperm production, Martina Franca donkeys

## Abstract

**Simple Summary:**

The Martina Franca Donkey is an endangered breed for which breeding recovery programs have been started. The animals involved in such projects should be characterized by high fertility, and testicular volume can be indicative of this. The study aimed to identify the most accurate formula for calculating testicular volume. The correlation between testicular volume and sperm production was also verified. Our results suggest that in Martina Franca jacks, testicular volume can be estimated with the formula V (cm^3^) = 33.57 × H − 56.57, and there is a significant correlation with sperm output. In conclusion, testicular size in healthy stallions is suggestive of good fertility and could be considered when choosing stallions to be approved for breeding.

**Abstract:**

Good sperm production is a notable requirement for subjects intended for reproduction, particularly in endangered species, and it has been demonstrated that in horse stallions, this is correlated to testicular volume. The present study, which involved Martina Franca jacks, aimed to determine whether, also in this endangered breed, there is a correlation between the total sperm number (TSN) and testicular volume. Testes were measured with both ultrasound and a caliper. Testicular volume was calculated using two different formulas: one representing the volume of an ellipsoid and one developed to describe round-shaped testicles. The average sperm concentration was 380.14 ± 254.58 × 10^6^/mL, while the average TSN was 16.34 ± 7.76 × 10^9^. Our findings evidenced a significant correlation (r > 0.75; *p* < 0.05) only between sperm production and the volume calculated with the formula V (cm^3^) = 33.57 × H − 56.57 for round-shaped testes. Moreover, significance was evidenced only for data obtained with ultrasound (VTs-us 315.03 ± 25.83 cm^3^) but not with caliper. In conclusion, testicular volume can be suggestive of good fertility in Martina Franca jacks; thus, this parameter could be considered when selecting breeding animals.

## 1. Introduction

Donkey breeding has been gaining interest all over the world for several decades, as it has zoo-technical value both for the production of milk and meat; in some cultures, donkey derivatives (particularly from the Dezhou donkey) are also used in traditional medicine [1,2]. Furthermore, in areas characterized by high animal biodiversity, protecting endangered native breeds, such as the Martina Franca donkey, is of paramount importance to preserve genetic variability [3,4]. The Martina Franca donkey breed is native to Puglia (Southern Italy). Being a large breed (adult males have an average weight of 400 kg), robust, and resistant, it has always been used for fieldwork. These qualities favored the use of Martina Franca jacks for the production of the mule, particularly used by the Italian Army for the transport of artillery and supplies during World War I [3]. Over the last century, however, the high mechanization of agriculture and the military industry has led to a decline in demand for the breed. For several decades, consequently, the number of animals allowed for reproduction has been less than 1000 [5] and is, therefore, currently considered an endangered species [4]. Another problem of the breed, resulting from the small number of subjects, is the high inbreeding, which makes it difficult to implement recovery plans [6].

A newfound interest in the Martina Franca donkey has led to a new increase in the demand for subjects, especially in Puglia, for use in tourist contexts such as trekking or farm holidays, but also, given their peaceful disposition, for pet therapy programs and animal-assisted interventions (AAIs) [7]. Finally, in central Italy, the mule is still used, particularly in rural territories and protected wooded areas, such as the “Gran Sasso and Monti della Laga National Park” in Abruzzo, for the transport of timber after logging operations. To produce this hybrid between jacks and mares, the Martina Franca stallion is often preferred to other breeds, due to its size and robustness, to mate workhorse mares, such as the Maremmana breed or the Italian Heavy Draft horses (CAITPR) [3].

A breeding recovery program and the preservation of genetic biodiversity have been underway for several years both in the Puglia region (in situ conservation projects) and in equine breeding centers, such as at the University of Teramo (ex situ conservation projects) [7]. The basis of such projects must always be a correct choice of breeding subject. As far as jacks are concerned, not only is the general health status and the absence of genital diseases important, but it is also important that they have good sperm production [2] to be able to cover more jennies in artificial insemination programs during the breeding season.

The testicular volume of a healthy stallion can represent, from this point of view, a good parameter for estimating its fertility. This is especially true for the donkey species, which has high spermatogenic efficiency, as it has a higher spermatid to Sertoli cell ratio than other species [8]. Among the parameters to take into consideration when evaluating a breeding subject are sperm volume and concentration, and the parameter that derives from these—total sperm number (TSN) [9]. Several studies have demonstrated a correlation between TSN and testicular volume in livestock and pet species. Starting from this correlation, daily sperm output (DSO) can be calculated, which is an estimate of the sperm that can be produced by testes of certain sizes [9]. Based on daily sperm production, the number of semen doses (refrigerated or frozen) that can be produced by a given subject with a single ejaculate can be calculated [2], and this information can be useful for managing mating plans efficiently in a breed recovery project. The correlation between TSN and testicular volume has been validated in the canine species [10], bovine species [11], and equine species [12,13,14]. Recent studies have also investigated this correlation for donkey stallions [2,15,16]. As with many aspects of donkey medicine, even regarding the calculation of testicular volume, the first studies borrowed the formulas used in equine practice [15]. However, recent publications [2] have highlighted inconsistencies mainly linked to the conformation of the asinine testes. In equine stallions, the testicles have a more elongated shape, similar to an ellipse, and the testicular volume in the horse can be calculated using the ellipsoid formula V (cm^3^) = 4/3π × H/2 × L/2 × W/2, with a good correlation with the TSN [17].

In jacks, however, the testicle has a more spherical shape, having a similar height and width, and this has also been demonstrated in the Martina Franca donkey [18]. Consequently, the volume estimate with the above formula does not find a significant correspondence with sperm production in the donkey [2,15]. Only in 2021, Magalhaes et al. [2] showed that in the Dezhou donkey, there is a correlation between sperm production and testicular volume, which was calculated using the formula V (cm^3^) = 33.57 × H − 56.57, which represents a regression equation obtained by El Wishy in a previous study [19] on donkey and horse testicular dimensions.

The present study aimed to verify whether sperm production can also be correlated to testicular volume in the Martina Franca jack and whether this can be better represented as sphere-shaped or ellipse-shaped. Based on these findings, the research also aimed to be able to suggest the evaluation of testicular size as a parameter for the approval of breeding jacks, as it is suggestive of good fertility.

## 2. Materials and Methods

### 2.1. Study Area and Animals

The present study was conducted in Chiareto, province of Teramo (Italy), latitude of 42°72′71″44 N, and longitude of 13°77′43″58 E. Semen samples were collected in May and June 2023 following the natural photoperiod.

The study involved 7 Martina Franca jacks, aged 4 to 7 years (average age 4.8 ± 1.2 years) and weighing between 370 and 440 kg (average weight 409.28 ± 25.88 kg). Throughout the breeding season, the animals were housed in paddocks with free access to hay and water; the food ration was supplemented daily with 1.5 kg of commercial feed for stallions. During the semen collection period for the present study, however, the subjects were moved to single boxes to isolate them (both from sight and smell) from jennies housed at the facility and to increase libido [20]. All the subjects, owned by the University of Teramo, had proven health and fertility by previous analysis. Moreover, at the beginning of the season, each subject underwent a breeding soundness evaluation and semen collection to confirm good health and fertility. Furthermore, all animals were regularly tested with negative results for mandatory screening analysis for infectious anemia [21] and sexually transmitted diseases (*Taylorella equigenitalis*, equine herpes virus, viral arteritis, dourine, West Nile) [22].

### 2.2. Semen Collection and Evaluation

The same group of experienced veterinarians and handlers always carried out semen collection. For the procedure, a Missouri model artificial vagina was used (one for each stallion, assigned at the beginning of the breeding season) in the presence of a dummy jenny in estrus.

Five semen collections for each subject occurred over ten days. The first four collections, carried out on alternate days, allowed the extra gonadal reserves to be eliminated and discarded. The subsequent semen sample, collected on the tenth day, underwent analysis. During collection, the reaction times of each subject were calculated, intended as the time elapsed between the first exposure to the mare and the achievement of a complete erection, followed by ejaculation [23]. Semen quality was assessed. The total volume (vol. tot) was assessed using a graduated cylinder immediately after collection. The gel-free volume (vol. gf) was calculated after filtration with sterile gauze in a graduated test tube. Concentration (conc.) and nonviable spermatozoa (death) were calculated with an automated sperm count system (Nucleo-Counter SP 100TM, ChemoMetec, Allerod, Denmark) [24]. Motility (total motile, progressive) and morphology (morph.) assessment were performed using a computerized semen analysis system (CASA IVOS II, Hamilton Thorne, Beverly, MA, USA) [25,26], with a standard setting provided by the manufacturer. Briefly, a sample was diluted 1:40 with an equine semen extender (INRA 96, IMV Technologies, Brooklyn Park, MN, USA) [27]; 5 µL was placed in a Makler chamber and analyzed using the following video setting: frame capture speed, 60 Hz; frame count, 30. The total number of ejaculated spermatozoa (total sperm number—TSN) was obtained by multiplying the concentration by the gel-free volume.

### 2.3. Testicular Measurements and Volume Calculation

Testes were measured with two different systems (scrotal caliper and ultrasound), always by the same experienced operator. To collect data, all subjects were adequately restrained in a stock with lateral access. No subject required sedation.

**Figure 1:**
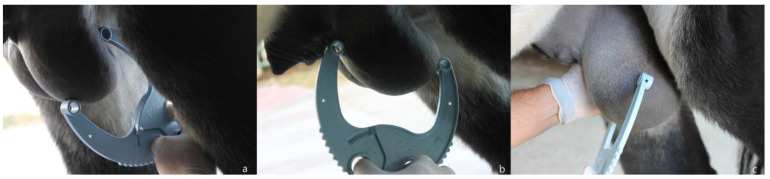
Scrotal measurement with caliper: (**a**) height (H-c); (**b**) length (L-c); (**c**) width (W-c)—the operator lifts the contralateral testicle with one hand to carry out a more accurate measurement. (*) legs of the caliper.

Scrotal measurements: Measurements were taken with a scrotal caliper (Stallion Scrotal Caliper, Animal Reproduction Systems) [14]. Height (right testicle height—Hr-c; left testicle height—Hl-c) was assessed by positioning the instrument lateral to the testis and placing the legs of the caliper ventrally and dorsally (Figure 1a). Length (right testicle length—Lr-c; left testicle length—Ll-c) was assessed by positioning the caliper ventral to the testis; legs were placed at the cranial and caudal poles, avoiding the tail of the epididymis (Figure 1b). Width (right testicle width—Wr-c; left testicle width—Wl-c) was assessed by positioning the instrument ventral to the gonad; the second testis was pushed toward the abdominal wall to allow for an accurate measurement, and the legs were positioned on the medial and lateral side of the testis (Figure 1c). For each dimension, three measurements were taken, using the average value obtained to calculate testicular volume.

**Figure 2:**
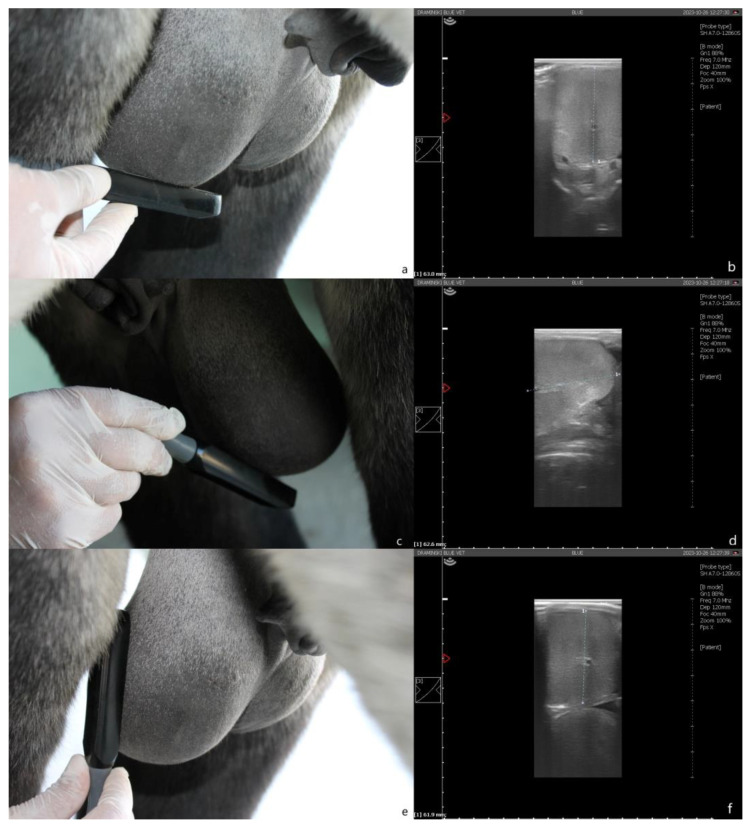
Ultrasound measurement. (**a**) Height assessment: the transducer is positioned ventral to the testis in a transverse position to the long axis. (**b**) Dorsal–ventral scan of the testis. (**c**) Length assessment: the transducer is positioned ventral to the testis with craniocaudal orientation (detail of the cranio–medial scan). (**d**) Cranio–medial scan of the testis. (**e**) Width assessment: the transducer is positioned lateral to the testis. (**f**) Latero–medial scan of the testis.

Ultrasound examination: Measurements were performed with a portable ultrasound device (Draminski Blue, Draminski Ultrasound Scanners, Sząbruk, Poland) using a 7 MHz linear probe (L60 probe, Draminski Ultrasound Scanners). Height (right testicle height—Hr-us; left testicle height—Hl-us) was assessed by positioning the probe ventral to the gonad in a transverse position to the long axis (Figure 2a,b). Length (right testis length—Lr-us; testis length—Ll-us) was assessed by positioning the probe ventral to the gonad, with a caudocranial orientation, scanning the gonad first in the cranio–medial segment and then in the medial–caudal one, to allow complete measurement (a marker was used to ensure complete measurement) (Figure 2c,d). Width (right testis width—Wr-us; left testis width—Wl-us) was assessed by positioning the probe lateral to the gonad, with dorsal–ventral orientation (Figure 2e,f). For each dimension, three measurements were taken, using the average value obtained to calculate the testicular volume.

For each testis, volume (V) was estimated using two different formulas, one representing a regression equation for round-shaped testes [2,19] (Vs) and one referring to the volume of an ellipsoid (Ve), as reported by Magalhaes et al. [2]:

Vs (cm^3^) = 33.57 × H − 56.57 (1)

Ve (cm^3^) = 4/3π × H/2 × L/2 × W/2 (2)

The total volume (VT) was deduced by adding the values of the right (Vr) and left (Vl) testes:

VTs (cm^3^) = Vls + Vrs (3)

VTe (cm^3^) = Vle + Vre (4)

Formulas were applied both for the measurements obtained with the caliper (VTe-cal; VTs-cal) and for those obtained with ultrasound (VTe-us; VTs-us).

### 2.4. Statistics

Statistical analysis was performed with JASP software (JASP, version 0.17, computer software, University of Amsterdam). Data normality was assessed with Shapiro–Wilk’s test. Testicular measures and volumes were compared with Student’s *t*-test (*p* < 0.001). Pearson correlation and linear regression were applied to TSN and volumes (VTe-cal; VTe-us; VTs-cal; VTs-us) (r > 0.5; *p* < 0.05).

## 3. Results

All animals completed the collection cycle positively. The average reaction time was about 13 min (ranges 5–40). Four-year-old subjects (D1–D4) showed average reaction times of 8 min and high libido, while the older subjects showed a tendency toward an increase and variability in reaction times. The fifth semen collection from each donkey was evaluated, and TSN was calculated. The average semen concentration was 380.14 ± 254.58 × 10^6^ spermatozoa/mL, ranging from 217 to 894 × 10^6^ spermatozoa/mL, while the average gel-free volume was 47.57 ± 18.72 mL, ranging from 30 to 85 mL. The average total sperm number (TSN), obtained by multiplying the concentration by the gel-free volume, was 16.34 ± 7.76 × 10^9^ (range 9.36 ± 26.82 × 10^9^). Sperm quality was assessed: total (range 82–97%) and progressive (range 54–80%) motility were acceptable for the breed. Table 1 shows the sperm analysis and quality assessment for all samples collected.

The mean values of height, length, and width obtained with the caliper were not significantly different from those obtained with ultrasound examination (*p* > 0.001). Length was significantly greater (*p* < 0.001) than height and width, both for measurements obtained with caliper and for those obtained with ultrasound. Table 2 highlights the average values obtained.

Different from individual dimensions, the volumes obtained using the Vs formula for round-shaped testes were significantly different (*p* < 0.001) from those obtained using the Ve formula for ellipse-shaped testes: in fact, VTs-cal was significantly different from VTe-cal (*p* < 0.001), and VTs-us was significantly different from VTe-us (*p* < 0.001). Comparing the measurement methods used, however, highlighted a statistically significant difference (*p* < 0.001) only between VTe-us and VTe-cal. There was, however, no significance (*p* = 0.003) between VTs-us and VTs-cal. Table 3 shows the average testicular volumes.

**Figure 3:**
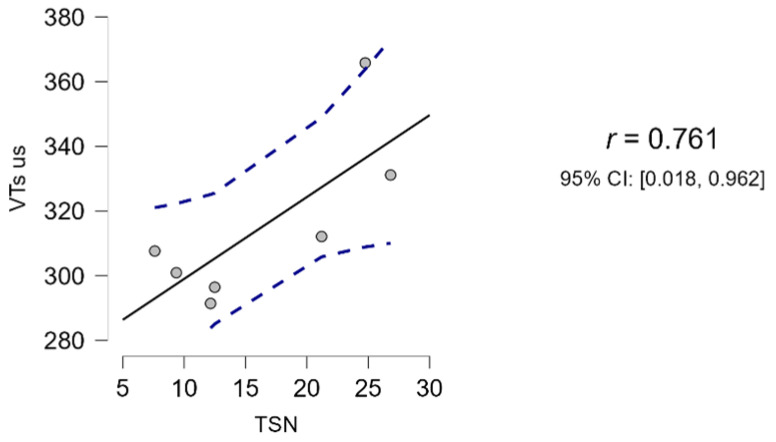
Regression line between sperm output (TSN) and testicular volume (VTs-us) (JASP, version 0.17, computer software, University of Amsterdam).

The average total testicular volume (VT) in Martina Franca jacks, obtained with ultrasound measurements and using the formula Vs (1), was 315.03 ± 25.83 cm^3^. This value was significantly correlated to the average TSN (16.34 ± 7.76 × 10^9^ spermatozoa), as shown in Figure 3. In fact, the Pearson correlation matrix only identified a significant correlation (r > 0.75; *p* < 0.05) between sperm number (TSN) and VTs-us. For all other volume calculation formulas (VTs-cal, VTe-cal, VTe-us), no correlation with sperm output was identified (r < 0.75; *p* > 0.05).

## 4. Discussion

Conservation and breeding programs should involve healthy, in line with standards, and highly fertile subjects. A method that has been used in the livestock sector for estimating fertility is the evaluation of testicular size and its correlation with daily sperm production [1]. Recently, such studies have also been carried out on the donkey population, both in breeds bred for commercial purposes, such as the Dezhou donkey [2], and in those at risk of extinction, such as the Ragusano donkey [15] and Miranda’s donkey [16]. The present study was carried out as part of study and recovery projects of an endangered breed to include testicular evaluation among the selection criteria for breeding jacks. In this context, selecting and approving jacks with good fertility would be optimal for their inclusion in recovery and breeding plans and to establish a jack-to-jennies ratio suited to the subject. Furthermore, in Martina Franca donkeys, previous studies have demonstrated that the testicular dimensions do not vary significantly during the year [18]; therefore, the present work was carried out during the reproductive season (May–June). Based on our previous studies, at these latitudes, the TSN should not vary significantly over the seasons even if, in the winter months, the reduction in daylight hours leads to a reduction in testosterone concentrations, resulting in a slight decrease in testicular volume and sperm concentration [18,23]. Liu et al. [1] also reported no significant changes in testicular volume and sperm concentration throughout the year.

Operating exclusively during the breeding season may have positively influenced libido and reaction times. The collections were always carried out in the presence of a jenny in full estrus, and in the Martina Franca breed, the spring and summer cycles are characterized by a longer duration of the estrus phase, with more evident behavioral manifestations (mouth-clapping, winking, urination) [7]. In the authors’ experience, a positive response to the jenny results in reduced reaction times. Prolonged reaction times, on the other hand, can influence semen evaluation, as they lead to the emission of ejaculates that are particularly rich in glandular secretions, with a high volume and reduced sperm concentration. The data observed reported reaction times of approximately 11–13 min in line with what has been described for the breed by Veronesi et al. [23], who referred to a value of 14 ± 9 min and a difference of only approximately 15 mL between the total volume and the gel-free volume. A reaction time range of 6 to 32 min has also been described by Gastal et al. [28] for the asinine species. In the authors’ experience, during the negative photoperiod, reaction times are longer, also due to the decreased estrus manifestations of the jennies. However, Magahlaes himself underlined that in the donkey species, sexual behavior could be widely variable from subject to subject and influenced by experience and environment [2]. The protocol described, in line with what was also reported by Rota et al. [20], involved the sheltering of stallions in closed boxes in anticipation of collections to keep the mares away and hidden from view. This increased testosterone and libido [20,23]. Reference [29] and the authors’ experience also found that maintaining a routine in stallion management (fixed route from paddock to the stable, same team of veterinarians and animal care staff) could also lead to stability in reaction times. Thus, managerial measures in conducting collections may have determined their success and the correct behavior of stallions.

Regarding the age of the subjects, both Veronesi et al. [23] and the data obtained from the study highlight that the increasing age of the stallions could lead to an increase in reaction times. This may seem at odds with previous data on the Pêga donkey, in which young animals showed longer reaction times [2], while the observed data showed slightly shorter times in younger subjects (4 years). A justification for this contrast could be found in the fact that, in donkeys, puberty is reached around 2 years of age [30], while complete sexual, physical, and behavioral maturity is reached only later. When choosing stallions to include in the study, subjects under 4 years of age were excluded, as, in the authors’ experience, they mostly showed disinterest, even toward the female in estrus, and it was not possible to complete the collection. These observations could support what Magahlaes et al. [2] reported on high reaction times in very young subjects and suggest that complete sexual maturity is only “late” in the donkey species and particularly in the Martina Franca breed.

Regarding the protocol chosen for semen collection, this differed from similar works [2,15], as the cited authors in both cases carried out sperm collection for 10 consecutive days. In Quartuccio’s work [15], the DSO was calculated starting from the ejaculates collected between the fourth and tenth day of collection, while Magahlaes et al. [2] considered the collections between days 8 and 10. In the work presented, semen was instead collected for 10 days on alternate days: to eliminate extragonadal reserves, four samples were taken in 8 days, while on the tenth day, the fifth sample was taken to estimate the TSN. This choice was justified by the fact that, on average, in the donkey species, it takes about 10 days to eliminate extragonadal reserves [31]. Nevertheless, the work of Quartuccio et al. [15] highlighted that starting from the fourth day, the average concentration remained quite stable; therefore, four samples in 8 days are sufficient to eliminate extragonadal reserves. Furthermore, sexual rest between one sample and another does not seem to influence semen quality, as the donkey species has very muscular ampulla glands that facilitate the elimination of nonejaculated spermatozoa in the urine [2,32]. Finally, the choice to carry out collections on alternate days may have determined the success of these. Although the cited papers do not report collection failures [2,15] in 10 days of consecutive collection, in donkeys, the variability in breeding behavior can also lead to a failure to collect [20,30]. Therefore, the nonintensive management of breeding subjects may be preferable to determine a standardization of the protocol and the success of semen collection.

As regards concentration and semen quality, the data obtained are in line with previous studies on the breed [18,25] and with what was reported for the similarly sized Dezhou donkey [2]. However, compared with the Andalusian donkey, which also has an average weight of around 400 kg, the semen material was more concentrated (380 × 10^6^ vs. 240 × 10^6^ spz/mL) and characterized by a slightly better average total and progressive motility (92–68% vs. 89–67%) [25]. This supports the choice of using the fifth sample of the proposed collection protocol to estimate sperm production, as it is representative of newly produced spermatozoa characterized by the best quality for the breed and the species.

Regarding testicular measurements, in the past, the caliper was considered the instrument of choice, also for the donkey species [31]. In recent years, however, studies have focused mainly on measurements carried out with ultrasound [1,2,15,16], as this tool is more accurate in measurement, allowing the evaluation of only the testicular parenchyma [14]. Furthermore, in the equine stallion, studies comparing the measurements obtained with the two different instruments highlighted that 2D ultrasound measurements are more accurate than those performed with the caliper [14] in estimating the volume of isolated testicles after orchiectomy. However, it should be underlined that in the cited paper, the caliper tended to underestimate testicular volume, while data obtained from the present study (Table 3) highlighted a higher estimate for caliper volume compared to ultrasound. This discrepancy is determined by the fact that Pricking et al. [14] measured isolated gonads after castration, so the data were not influenced by the presence of scrotum. The measurements carried out in intact subjects, however, were affected by this, albeit minimal, “thickness” in the case of measurement with the caliper. Measurements taken with ultrasound were not affected. Another consideration is that the discrepancies between the two measuring systems were not significant if the single measures of length, width, and height were considered (Table 2) but became significant when these parameters were added together and both testes were considered (Table 3). This is particularly evident when considering the formula for an ellipsoid, which includes all three dimensions. In this case, the presented research highlighted a significant difference between volume measurement with caliper and ultrasound. The volume calculation with the equation for round-shaped testes, on the other hand, was less affected by the greater or lesser accuracy of the measurement method, as it only considered height. The data obtained showed that although the average volumes obtained with the caliper were higher, the difference was not statistically significant (Table 3).

Regarding the choice of the most appropriate formula for calculating testicular volume in the donkey species, many authors have used the one that describes an ellipsoid [15,18,33]. However, in the only two works that, to the authors’ knowledge, have investigated its correlation with daily sperm production [2,15], the results obtained were not statistically significant. A study on the Ragusano donkey reported a linear correlation with an R^2^ of less than 0.5, which the authors themselves considered unacceptable [15]. A study on the Dezhou donkey also highlighted that there was no correlation between the volumes estimated with the ellipse formula, TSN, and expected DSO [2]. However, the same study reported a strongly positive correlation (r = 0.75, *p* < 0.05) between TSN (defined by the authors as actual DSO), expected DSO, and volume calculated using a formula for round-shaped testes [2,19]. In the present work, the same formulas described by Magahlaes et al. [2] were used, confirming the latter’s findings. Indeed, the study demonstrated a significant correlation (r > 0.75; *p* < 0.05) (Figure 3) between TSN and VTs-us and no correlation with other volumes. The data presented, compared with those of Quartuccio et al. and Magahlaes et al. [2,15], confirm that in donkeys, testicular volume is best represented by the equation for round-shaped testes. This finding is justified, as in donkeys, particularly the Martina Franca breed, testicular height and width were similar (about 6–8 cm), while length differed considerably from the others (Table 2). Other studies reported in the literature confirmed these relationships between testicular dimensions, both for the breed in question [18] and for others of similar size [2,15]. Therefore, the formula Vs (cm^3^) = 33.57 × H − 56.57 allows an accurate estimate of the volume, determining, as for other species, a significant correlation with the total sperm number. Further studies are needed on a greater number of animals and semen samples to confirm the data obtained and to determine a formula to predict the daily sperm output in Martina Franca jacks. In fact, in this protocol, only one semen sample per subject was considered to verify which of the proposed formulas could be best correlated to the TSN.

## 5. Conclusions

The Martina Franca donkey is one of the Italian endangered breeds whose genetic heritage should be preserved to protect biodiversity. Breed recovery programs cannot ignore a careful choice of animals, which should respect the expected standards but also be healthy and fertile. The data obtained in the present study highlighted that, in Martina Franca jacks, the evaluation of testicular volume is suggestive of good fertility since it is correlated to sperm production. This volume can be calculated with the formula Vs (cm^3^) = 33.57 × H − 56.57, where H is the height of the testis, measured with ultrasound for greater accuracy. In conclusion, the presented study suggests that testicular volume may be considered among the evaluation criteria for the approval of breeding donkeys. Furthermore, by evaluating testicular volume routinely in subjects included in breeding programs, it will be possible to confirm the correlation between volume and sperm production and the repeatability of results and to perfect the volume equation for the Martina Franca breed.

## Figures and Tables

**Table 1:** Sperm analysis for each subject and mean ± standard deviation obtained.

	D1	D2	D3	D4	D5	D6	D7	Mean ± St. Dev.
Reaction time (min)	5	5	13	11	6	11	40	13 ± 12.34
Total volume (ml)	65	70	60	46	60	100	55	65.14 ± 17.13
Gel-free volume (ml)	56	47	35	35	30	85	45	47.57 ± 18.72
Concentration (×10^6^ spz/mL)	217	265.8	217.3	267.5	894	249.4	550	380.14 ± 254.58
Motile (%)	97	82.6	97.2	92	88.7	91	96	92.07 ± 5.29
Progressive (%)	64.5	72.5	73.6	54.6	70.5	63	80	68.38 ± 8.34
Death (×10^6^ spz/mL)	31.55	42.21	13.06	36.18	36.8	16.48	53	32.75 ± 14.03
Morph. (%)	98.5	88.8	99.2	95.6	98.5	92	99	95.94 ± 4.07
TSN (×10^9^)	12.15	12.49	7.60	9.36	26.82	21.19	24.75	16.34 ± 7.76

TSN, total sperm number. D1–D7 indicates jacks included in the study.

**Table 2:** Average dimensions (mean ± st. dev.), expressed in cm, of the testicular measurements obtained with caliper and ultrasound.

	Caliper (c)		Ultrasound (us)	
	Left Testis (l)	Right Testis (r)	Left Testis (l)	Right Testis (r)
Length (L)	10.99 ± 0.89 ^a^	11.10 ± 0.81 ^a^	10.49 ± 0.37 ^a^	10.52 ± 0.73 ^a^
Width (W)	6.2 ± 0.45 ^b^	6.78 ± 0.28 ^b^	5.89 ± 0.17 ^b^	6.13 ± 0.62 ^b^
Height (H)	7.92 ± 1.02 ^b^	7.83 ± 0.43 ^b^	6.30 ± 0.40 ^b^	6.45 ± 0.57 ^b^

Different superscripts (^ab^) within columns indicate statistically significant differences (*p* < 0.001) (Student’s *t*-test).

**Table 3:** Average dimensions (mean ± st. dev.), expressed in cm^3^, and range of total testicular volumes (VTs) obtained from measurements with caliper (cal) and ultrasound (us).

	VTs-cal	VTs-us	VTe-cal	VTe-us
Average dimensions	415.98 ± 42.25	315.03 ± 25.83	595.51 ± 91.89	257.19 ± 31.27
Range of testicular volumes	(362.43–497.83)	(291.37–365.79)	(485.04–755.79)	(220.91–316.37)

Vs: 33.57 × H − 56.57. VTs: Vs right + Vs left. Ve: 4/3π × H/2 × L/2 × W/2. VTe: Ve right + Ve left.

## Data Availability

Data are contained within the article.
